# Coexisting mechanisms of luminogenesis in pancreatic cancer-derived organoids

**DOI:** 10.1016/j.isci.2024.110299

**Published:** 2024-06-18

**Authors:** Samuel J. Randriamanantsoa, Marion K. Raich, Dieter Saur, Maximilian Reichert, Andreas R. Bausch

**Affiliations:** 1 Technical University of Munich, TUM School of Natural Sciences, Department of Bioscience, Chair for Cellular Biophysics E27, 85748 Garching, Germany; 2 Technical University of Munich, Center for Functional Protein Assemblies (CPA), 85748 Garching, Germany; 3 Technical University of Munich, Center for Organoid Systems and Tissue Engineering (COS), 85748 Garching, Germany; 4 Technical University of Munich, School of Medicine, Klinikum rechts der Isar, Medical Clinic and Polyclinic II, 81675 Munich, Germany; 5 German Cancer Research Center (DKFZ), German Cancer Consortium (DKTK), Partner site Munich, 69120 Heidelberg, Germany; 6 Technical University of Munich, Klinikum rechts der Isar, Medical Clinic and Polyclinic II, Translational Pancreatic Cancer Research Center, 81675 Munich, Germany

**Keywords:** Biological sciences, Cancer, Cell biology, Organoids, Morphogenesis

## Abstract

Lumens are crucial features of the tissue architecture in both the healthy exocrine pancreas, where ducts shuttle enzymes from the acini to the intestine, and in the precancerous lesions of the highly lethal pancreatic ductal adenocarcinoma (PDAC), similarly displaying lumens that can further develop into cyst-like structures. Branched pancreatic-cancer derived organoids capture key architectural features of both the healthy and diseased pancreas, including lumens. However, their transition from a solid mass of cells to a hollow tissue remains insufficiently explored. Here, we show that organoids display two orthogonal but complementary lumen formation mechanisms: one relying on fluid intake for multiple microlumen nucleation, swelling and fusion, and the other involving the death of a central cell population, thereby hollowing out cavities. These results shed further light on the processes of luminogenesis, deepening our understanding of the early formation of PDAC precancerous lesions, including cystic neoplasia.

## Highlights

• Murine PDAC organoids form “clear” and “dark” lumens via two different mechanisms
• “Clear” lumens form via fluid intake inducing microlumen nucleation and fusion
• “Dark” lumens form via the death of a central cell population, hollowing branches
• Complex structures allow the coexistence of both mechanisms in single organoids

## Graphical abstract

**Figure:**
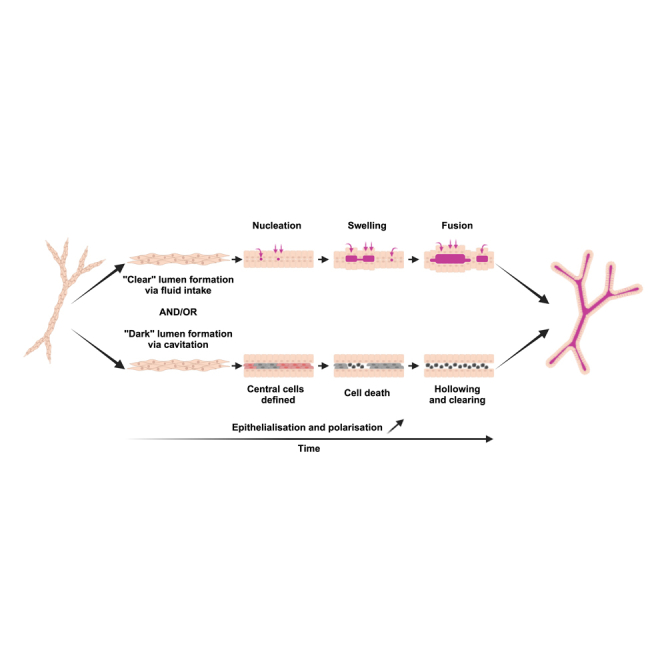

## Introduction

Pancreatic ductal adenocarcinoma (PDAC), a highly lethal disease originating in the exocrine pancreas, owes its elevated mortality rates, for a large part, to the lack of early markers for timely diagnosis, and a poor understanding of the disease’s progression.^1^

Indeed, only 20% of the detected pancreatic cancer cases are diagnosed early enough to be eligible for resection, the only available curative option.^2^ Addressing the disease at the pre-invasive stage could therefore bring about major improvements in patient prognosis. PDAC has been reported to arise from a variety of precancerous lesions, notably pancreatic intraepithelial neoplasia (PanIN) and intraductal papillary mucinous neoplasia (IPMN)—which both present as mucin-producing epithelia involving branches and ducts—and mucinous cystic neoplasia (MCN), which adopt a cystic shape.^3^^,^^4^^,^^5^^,^^6^^,^^7^ The mechanisms that lead to the apparition and development of these lesions, and the dynamics of progression to the invasive form of PDAC, remain unclear.

Organoids, three-dimensional cell cultures recapitulating key features of specific organs or tumors offer a promising platform to study the dynamics of cancer progression *in vitro*, and determine hallmarks of early tumor formation to improve diagnosis capabilities.^2^ A prior study^8^ demonstrated that PDAC organoids mimicked both healthy and cancerous pancreatic architectures, from their branched architectures displaying terminal buds, to the formation of duct-like structures. Here, we investigate the dynamics of luminogenesis processes by which those duct-like structures emerge.

Previous reports have evidenced that the formation of biological *de novo* lumens from rod-like structures often involves one of two classes of processes^9^: processes that rely on cell death to clear a space inside the rod-like structure to transform it into a tube^10^^,^^11^^,^^12^—so-called “cavitation” processes—and processes that do not rely on cell death but rather on cellular rearrangements to generate a lumen.^13^^,^^14^^,^^15^ Rarely, systems such as 3D Madin-Derby canine kidney (MDCK) cysts, have been reported to switch between non-apoptotic and apoptotic mechanisms to form lumens, when their culture conditions are heavily modified (depending on whether they were grown in Matrigel or collagen, and depending on whether cell polarity was promoted).^16^

We report that lumen formation in PDAC organoids can occur both through a cell death-free process involving fluid intake and through a process involving the apoptosis of a central cell population, two distinct mechanisms that are seldom observed together within the same system under the same culture conditions.^9^^,^^17^^,^^18^ We highlight the progressive epithelialization and polarization occurring during lumen formation, and find that mucin 1 (MUC1), a marker of pancreatic lumens in healthy and diseased tissues,^4^^,^^5^^,^^19^ concomitantly lines the apical side, further underscoring the recapitulating capabilities of our system. Our study therefore contributes to the understanding of the luminogenesis processes involved in the morphology of precancerous lesions-like structures.

## Results

### The existence of clear and dark lumens suggests different processes of luminogenesis at play

Over the course of their growth, branched PDAC organoids have been reported to develop a single seamless lumen connecting the network of branches, via the formation of microlumens.^8^

**Figure 1:**
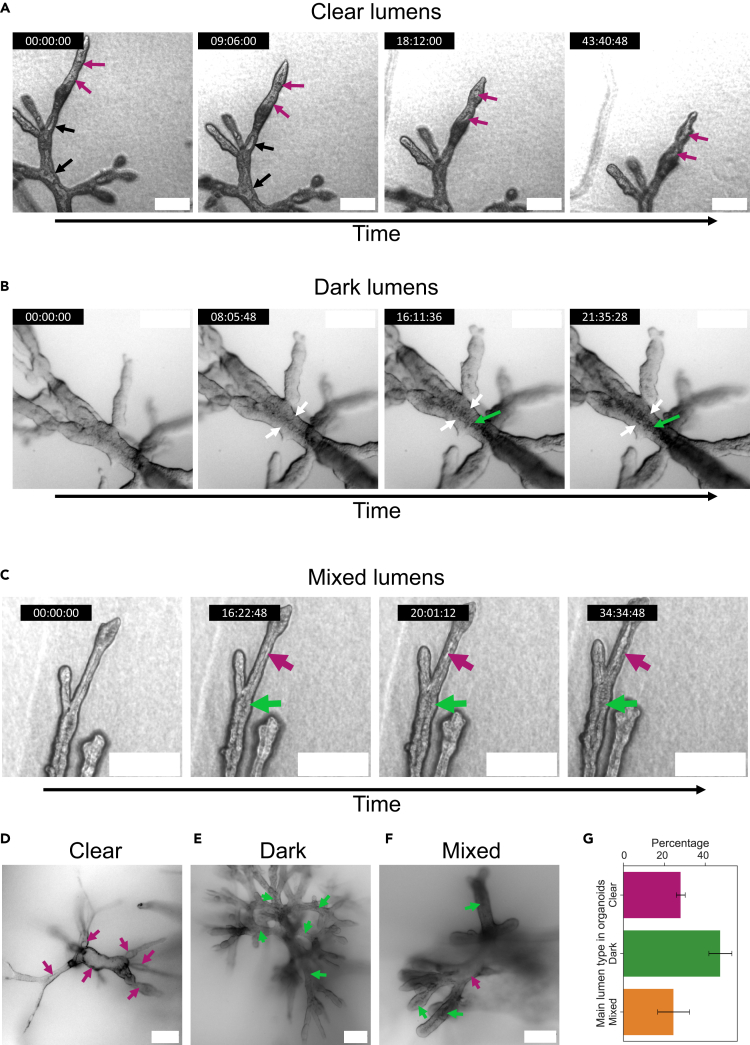
Organoids form “clear” and “dark” cavities (A) Close-up view of branches displaying the formation of so-called “clear” lumens, cavities highly translucent in bright field microscopy. Black arrows indicate cavities that had already nucleated and swollen before observation started, while magenta arrows indicate cavities newly nucleated. (B) Close-up view of branches displaying the formation of so-called “dark” lumens, cavities poorly translucent in bright field microscopy. White arrows indicate the epithelial-like wall surrounding the lumen, while green arrows indicate the core of the branch, progressively becoming darker and eventually giving rise to a cavity. (C) Clear (magenta arrow) and dark lumen (green arrow) coexisting next to each other within a branch. (D) Entire organoid with predominantly clear lumens. (E) Entire organoid with predominantly dark lumens. (F) Entire organoid with both clear and dark lumens (resp. magenta and green arrows). (G) Average percentage of organoids with predominantly clear (*N* = 16/57), dark (*N* = 27/57), and mixed lumens (*N* = 14/57 organoids). Error bars indicate the standard error of the weighted mean. Scale bars: 200 μm.

Using high-content long-term imaging to observe a large number of organoids prior and during their lumen formation phases, we identified two types of microlumens that appeared distinct from each other in transmitted light microscopy (Figures 1A, 1B, 1D, and 1E, Videos S1 and S2), suggesting different mechanistic routes toward the formation of a single lumen. The first type of microlumens appeared highly translucent in bright field and was therefore referred to as “clear microlumens”, whereas the second type, that appeared much less bright and filled with cells or cell debris, was referred to as “dark microlumens”. While some organoids exhibited preferentially one microlumen type or the other (28.07% “clear” and 47.37% “dark” out of *N* = 57 organoids Figure 1G), we report the existence of mixed organoid phenotypes that displayed both clear and dark lumens (24.56% “mixed” out of *N* = 57 organoids) (Figures 1C, 1F, and 1G).

**Figure 2:**
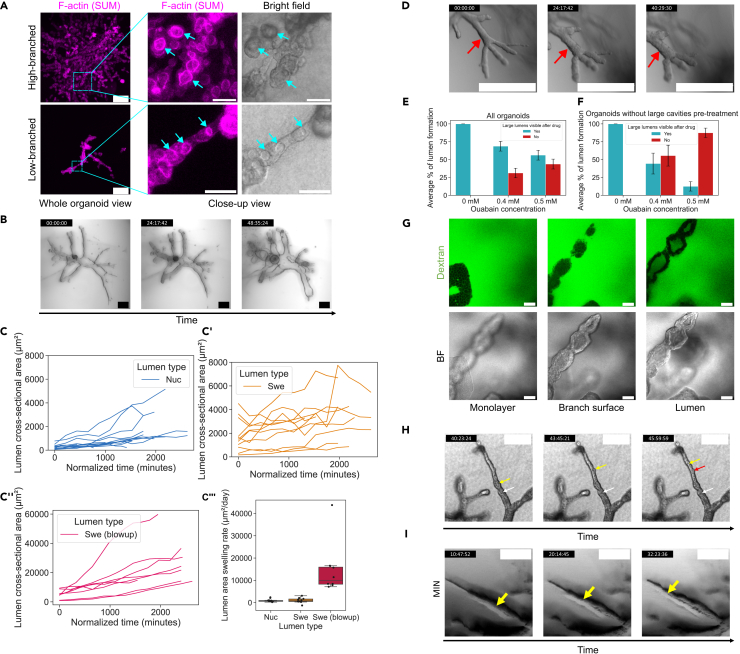
Fluid intake and fluid transfer contribute to clear lumen formation (A) Phalloidin staining of high-branched and low-branched organoids with “clear” microlumens. Summed slice projection shows F-actin lining the microlumens as indicated by the blue arrows. (B) Organoid displaying an extreme cavity swelling behavior during the lumen formation phase. Evolution of lumens’ cross-sectional area over time in (C) “Nuc” (n = 14 lumens, *N* = 5 organoids), (C’) “Swe” (n = 11 lumens, *N* = 4 organoids), (C”) blowing up (n = 8 lumens, *N* = 3 organoids) cases, and estimated rates of swelling shown in boxplots (C”’). Boxplots indicate the interquartile range (IQR, rectangle), the median (line), and 1.5 times the IQR (whiskers). Tracks with fusion events between neighboring cavities are excluded from the computation of the swelling rates. (D) Time-lapse showing that treatment with 0.4 mM ouabain at the Thickening stage, prior to lumen apparition, can prevent further lumen formation. The red arrow indicates the place where a lumen would normally have been expected to form in untreated conditions after more than 40 h. Drug is added at time point 00:00:00, on a Day 11 organoid. Lumen formation post-treatment is quantified in E and F. (E) considers organoids both with and without already formed cavities pre-treatment (0 mM, *N* = 18; 0.4 mM, *N* = 16; 0.5 mM, *N* = 16 organoids), whereas (F) considers only organoids that did not display any lumen before treatment (0 mM, *N* = 8; 0.4 mM, *N* = 9; 0.5 mM, *N* = 8 organoids). Error bars in E and F indicate the standard error of the weighted mean. (G) Confocal slices showing Dextran Alexa 488 incorporated at the cell-cell junctions in a monolayer, at the branch surface, and inside the lumen. Dextran in the lumen was detectable 315 min after the addition. (H) Time-lapse showing the propagation of fluid along a fault line in a branch, in a process reminiscent of hydraulic fracturing. As it propagates, the fluid creates transient microlumens that grow then deflate. The white arrow indicates the position of the fluid’s source, the yellow indicates the position of the currently growing microlumen, and the red indicates the position of a deflating microlumen. (I) Time-lapse showing the propagation of a fracture (yellow arrow), along the branch longitudinal axis due to fluid intake. Note that the minimum intensity z-projection causes the inside of the lumen to appear darker than it is in reality. Scale bars: A (whole organoid), B, H, and I 200 μm; D 500 μm; A (close-up), G 50 μm. Fluorescence images in A,B,E: confocal summed projections; in F: confocal slices. Bright field images in A, D, G, and H: plane; in B and I: minimum projection. See also Figures S1 and S3.

Video S1. Clear lumen formation Predominant clear lumen formation in a PDAC organoid during the Lumen formation phase (day 10–13). Bright field plane, drift-corrected. Time-lapse imaging consists of an image taken every 80 min and 59 s, and is displayed at 20 fps. Time (hh:mm:ss). See also Figures 1 and 2.

**Figure 3:**
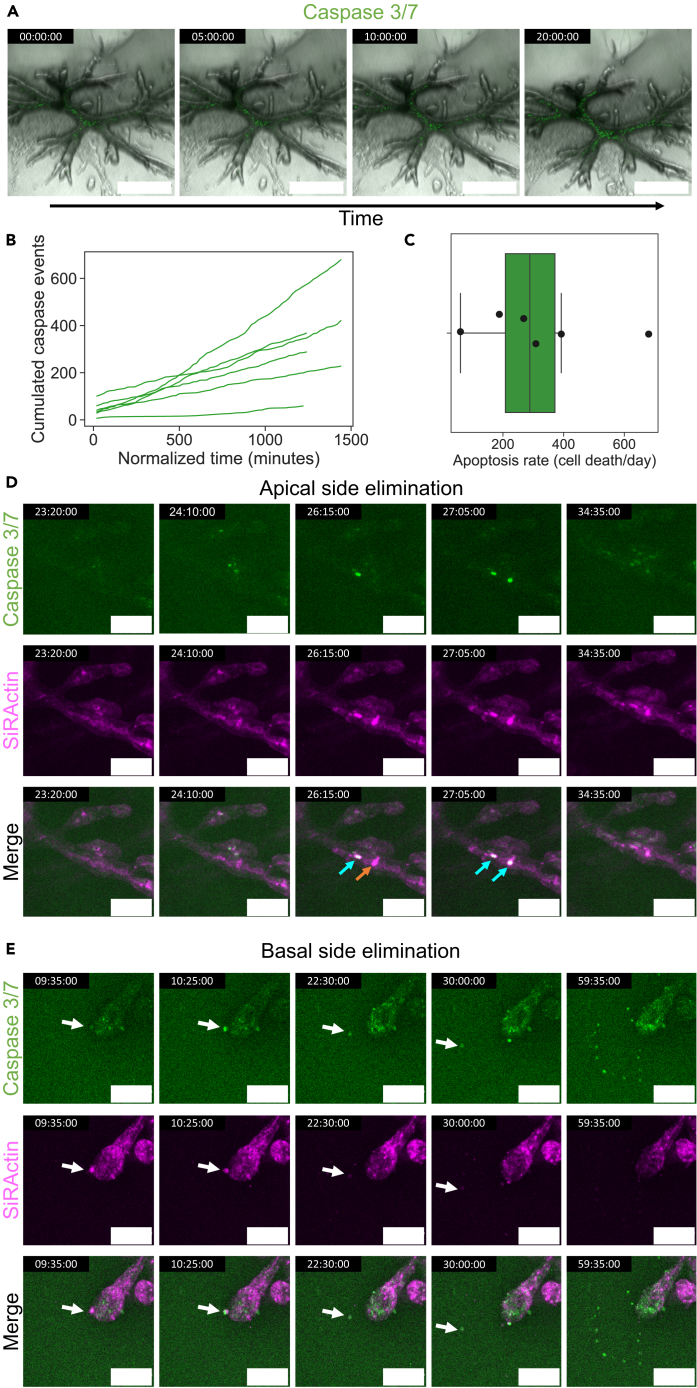
Apoptosis contributes to dark lumen formation via cavitation, and to cell elimination in the apical and basal directions (A) Summed slice projection time-lapse of an organoid labeled with NucView caspase-3 enzyme substrate showing apoptotic events (in green) leading to the formation of dark lumens in the core and in the branches. (B) Cumulated caspase events detected in whole organoids during dark lumen formation (*N* = 6 organoids), and corresponding apoptosis rate. (C) Boxplot indicates the interquartile range (IQR, rectangle), the median (line), and 1.5 times the IQR (whiskers). (D) Time-lapse of a cell elimination event, from the epithelium to the apical side, post-lumen formation, visible through caspase 3/7 activity (green) and strong increase in the actin signal (magenta, labeled with SiR-actin). Orange arrows indicate cells being eliminated displaying an increased actin signal but no caspase signal yet, while cyan arrows indicate the presence of both an increased actin signal and of caspase activity. (E) Time-lapse of a cell elimination event, from the epithelium to the basal side, post-lumen formation, visible through caspase 3/7 activity (green) and strong increase in the actin signal (magenta, labeled with SiR-actin). The white arrow tracks the same cell over time. Scale bars: A: 500 μm; D and E: 100 μm. Fluorescent images in A, D, and E are confocal summed projections. See also Figures S1–S3.

Video S2. Dark lumen formation Dark lumen formation in a PDAC organoid during the Lumen formation phase (day 10–13). Bright field planes, drift-corrected. Time-lapse imaging consists of an image taken every 80 min and 58 s, and is displayed at 10 fps. Time (hh:mm:ss). See also Figures 1 and 3.

This indicates that the processes leading to the formation of those lumens are not mutually exclusive at the organoid level, and can even coexist within the same branch (Figure 1C).

### Focusing on clear lumens: A fluid-based lumen formation mechanism

Given the translucid appearance of the clear microlumens, we hypothesized that fluid intake could be the major driver for the apparition of those cavities.

We first observed those clear cavities in organoids of various sizes and branch numbers by staining against actin, which is lining the apical surface, and bright field images, indicating that clear lumen formation may proceed irrespective of organoid phenotypes (Figure 2A). Through live imaging data, we tracked and measured the swelling profiles of both nucleating lumens and already established swelling lumens at the time of recording start (respectively, “Nuc” and “Swe” lumens in Figures 2C, 2C’, S1A, see STAR Methods for the distinction between the types). Measurements of the swelling rates for cross-sectional areas appeared overlapping for the nucleating and swelling lumens, with respective median rates of 719 μm^2^/day and 792 μm^2^/day (Figure 2C”’).

In addition, live imaging dozens of organoids in parallel allowed us to identify that this clear lumen-forming mechanism was not restricted to microlumen formation, but could also form very large macrolumens in a subpopulation of organoids that displayed a “blowup” behavior (5 out of 57 lumen-forming organoids [8.77%]), with large translucent lumens swelling (Figure 2B and Video S3). These blowup phenotypes possessed an area expansion rate superior by an order of magnitude, despite being, pre-lumen formation, morphologically similar to the non-blowup phenotypes, reaching a median rate of 9918.67 μm^2^/day (Figures 2C” and 2C”’).

Video S3. Lumen blowup Extreme example of clear lumen formation in a “blowing up” organoid during the Lumen formation phase (day 10–13). Minimum intensity projection of bright-field imaging, drift-corrected. Time-lapse imaging consists of an image taken every 80 min and 59 s and is displayed at 20 fps. Time (hh:mm:ss). See also Figure 2.

The architecture of the non-blowup phenotypes thus appears closer morphologically to PanIN lesions, whereas blowup phenotypes may be reminiscent of the architectural features of other subpopulations of precancerous lesions such as IPMN or MCN that involve larger duct dilation.^3^^,^^6^^,^^7^

Indicative of the importance of fluid intake in lumen nucleation and growth, we found that by applying ouabain (Tocris, #1076/100), a Na^+^/K^+^ ATPase inhibitor,^13^^,^^20^^,^^21^ prior to any nucleation event, we could prevent lumen formation in a population of organoids (Figures 2D–2F). We noted that while ouabain treatment appeared to impair lumen development if administered before nucleation events, organoids that had already started developing clear and dark lumens could continue their luminogenesis (Figures 2E, S1B, and S1C). This suggests that Na^+^/K^+^ pumps play a key role in facilitating the nucleation events, but that their importance in lumen expansion may decrease post-lumen opening.

By adding fluorescent dextran (3000 MW Alexa 488, anionic, D34682 Thermofisher) to the culture medium during the lumen formation phase, we found an uptake of dextran in the cavities concomitant with fluid intake, and an accumulation of dextran in the cell-cell junctions, suggesting a contribution of a paracellular route for fluid intake in luminogenesis (Figure 2G).

Moreover, we could observe transfers of fluid along a central fault line of the branches, leading to the apparition of microlumens or elongating an already open lumen, through fluid propagation from an already established cavity, thereby separating adjacent cells along this longitudinal axis (Figures 2H and 2I, and Video S4). This process appears highly reminiscent of hydraulic fracturing mechanisms, where fluid propagating through cracks is able to form cavities.^22^

Video S4. Propagation of fluid through a fault line Propagation of fluid through a fault line in the center of a branch, reminiscent of hydraulic fracturing, in a PDAC organoid during the Thickening and Lumen formation phases (day 9–12). The white arrow indicates the cavity at the base of the branch, the yellow arrow indicates a nucleating microlumen and the current position of the fluid “pulse”, while the red arrow marks a deflating microlumen, indicating that the fluid “pulse” is exiting the cavity. Bright field plane, drift-corrected. Time-lapse imaging consists of an image taken every 67 min and 19 s, and is displayed at 5 fps. Time (hh:mm:ss). See also Figure 2.

### Focusing on dark lumens: A cell death-based lumen formation mechanism

In contrast to “clear” lumens, “dark” lumens, appeared to be less translucent in transmitted light microscopy (Figure 1B).

Previous observations on PDAC organoids had evidenced, post-lumen formation, the presence of apoptotic cells in the cavities, displaying a cleaved caspase-3 signal, as well as processes of cell extrusions from what becomes the mono-layered epithelium that lines the lumen.^8^ However, whether apoptosis and extrusion were contributing to lumen formation or were byproducts of it, remained unclear.

**Figure 4:**
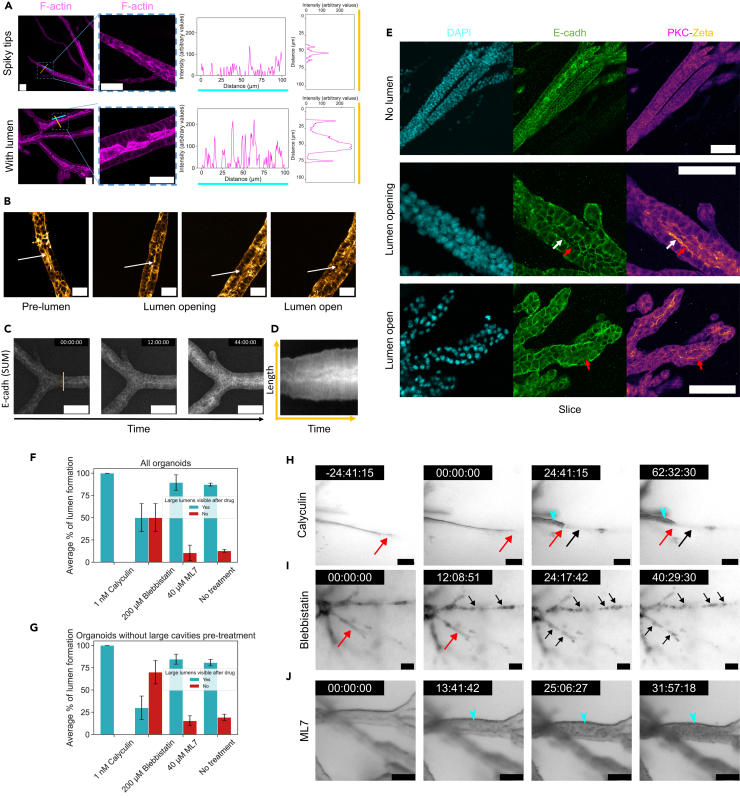
Organoids undergo progressive epithelialization and polarization along the central (fault) line (A) From left to right: F-actin organization in a Phalloidin Alexa 633-stained organoid, closeup, longitudinal actin intensity profile along the cyan line, transversal actin intensity profile along the orange line. Top: organoid pre-Thickening. Bottom: organoid post-Thickening. (B) From left to right: Distribution fluorescent signal in organoids, overexpressing GFP-tagged non-muscle myosin IIa, pre-, during, and post-lumen opening. White arrow indicates the central line of the branch along which the lumen nucleate. (C) Time-lapse of E-cadherin distribution in an endogeneously labelled organoid during the Thickening phase, showing a global increase. The corresponding kymograph taken along the orange line is shown in (D). (E) Top to bottom: Evolution of the E-cadherin and PKC-ζ distribution pre-, during, and post-lumen opening. The white arrow indicates a cell where the apical side still expresses both E-cadherin and PKC-ζ, while the red arrow indicates a cell where the apical side is polarized with PKC-ζ but has lost its E-cadherin signal. Microscopy images shown are all confocal slices except for C which shows summed projections. (F) Average percentage of lumen formation in organoids treated at the thickening-to-lumen formation phase with calyculin, blebbistatin, ML7, or untreated (resp. *N* = 8, 14, 19, 39 organoids) at various concentrations. Both organoids with and without large cavities prior to drug addition are quantified in F. A similar analysis restricted to organoids that did not display large lumens before drug addition is shown in G (with resp. *N* = 7, 10, 13, 26 organoids). Further concentrations are explored in Figure S4. Error bars indicate the standard error of the weighted mean in F,G. Drug is added at time point 00:00:00 in H–J. (H) Calyculin treatment—shown here at 1 nM—on a phenotype still in extension at drug addition time, stops the branch extension and trigger the formation of bud-like structures as the increased contractility leads to a rupture between tip cells that remain attached to collagen fibers in front of them and cells at the back (red arrow) that retract along the path previously digested in collagen (black arrow). The cyan arrowhead indicates a lumen forming post treatment. (I) Blebbistatin treatment—shown here at 150 μM—can trigger the fragmentation of branches in thin-branched phenotypes, with individual cells rounding up. Red arrows indicate intact branches, black arrows indicate broken branches. (J) ML7 treatment—shown here at 50 μM—can trigger a rapid filling of clear lumens, with dark cells, as indicated by the cyan arrowhead. Scale bars: A and B 50 μm; C, E, I, and J 100 μm; H 200 μm. See also Figures S3 and S4.

To investigate, we performed live imaging during the period spanning the thickening and the lumen formation phases, using fluorescent caspase-3 activity reporters (#10402 and #10406 Biotium). We could observe that in the core of organoids and in the center of their branches originally devoid of lumens, the darkening was correlated with multiple cell death events, that appeared to occur largely “in place” (i.e., without extrusion), evidenced by the increasing cleaved caspase-3 signal, corresponding to hundreds of cells dying in a day (Figures 3A–3C and S2, and Video S5). The central position of those apoptotic cells, sandwiched between the columnar cells forming the epithelium-like layer enclosing the future lumen, appeared to correlate with the position of elongated cells surrounded by cells displaying an increasingly strong apical E-cadherin signal (Figures 4C–4E). This central cell population, after detaching from the lining epithelium, could then be shuttled in the luminal network by fluid transfers, and ends up degrading over time (Figure S2).

Video S5. Cell death event labelling Composite movie showing Caspase 3/7 activity (green) labeled using 5 μM NucView 488, and bright field, during dark lumen formation in a PDAC organoid. Summed slice projection, imaged via confocal microscopy. Time-lapse imaging consists of an image taken every 20 min, and is displayed at 20 fps. Time (hh:mm:ss). See also Figure 3.

This indicates that in addition to the previously described fluid-intake based mechanism, hollowing the branches through cavitation is another option for lumen opening in PDAC organoids (Figures S1C and S1D).

Following cavity opening, we could observe further cell death, this time mostly resulting from elimination events in the epithelium, which strikingly, could occur both toward the apical side (Figure 3D and Video S6) but also in the basal direction (Figure 3E and Video S7) even when an apical lumen existed (Figure S1E). Given the plateauing in cell numbers in the organoids and the decrease in the proliferation-capable population during the thickening and lumen formation phases,^8^ we hypothesize that these extrusion events might contribute to tissue homeostasis (see Discussion).

Video S6. Cell elimination event in the apical direction Composite movie with Caspase 3/7 activity (green) labeled using 1:500 (v:v) NucView 530, and F-actin labeled using 100 nM SiR-actin, showing cell elimination events toward the apical side, in a PDAC organoid post Lumen formation (day 14–16). Summed slice projection, imaged via confocal microscopy. Time-lapse imaging consists of an image taken every 25 min, and is displayed at 20 fps. Time (hh:mm:ss). See also Figure 3.

Video S7. Cell elimination event in the basal direction Composite movie with Caspase 3/7 activity (green) labeled using 1:500 (v:v) NucView 530, and F-actin labeled using 100 nM SiR-actin, showing cell elimination events toward the basal side, in a PDAC organoid post Lumen formation (day 14–17). Summed slice projection, imaged via confocal microscopy. Time-lapse imaging consists of an image taken every 25 min, and is displayed at 20 fps. Time (hh:mm:ss). See also Figure 3.

### Focusing on the center of branches: Epithelialization and polarization accompany the lumen opening

We investigated the changes in tissue mechanics-, cell-cell adhesion-, and cell polarity-associated proteins, to understand the mechanisms required for the transition of branches from a solid rod shape to a hollow one, the positioning of future lumens, the separation of adjacent cell walls, and the maintenance of tissue integrity upon fluid intake.

Having observed the existence of a central fault line in the clear lumens case, and the targeted death of a central population in the case of dark lumens, we focused on the protein distribution in the middle of branches.

Investigating the cytoskeleton, specifically acto-myosin, as one part of tissue-mechanics, we observed high levels of F-actin and non-muscle myosin IIa along a central line in the branches prior to lumen nucleation (Figures 4A, 4B, and S3). As lumens nucleate, the F-actin signal remained intense at the apical side, delineating the cavities, while the signal of non-muscle myosin IIa progressively decayed.

During clear lumen formation, fluid intake appeared to separate adjacent cells in branches, therefore we aimed to determine if cavity opening resulted from a complete loss of cell-cell adhesion at the nascent apical side, or if cell-cell adhesion was preserved, thereby suggesting that fluid intake was able to overcome adhesive interactions. To this end, we looked at the dynamics of E-cadherin expression using an endogenously labeled cell line, and observed that prior to lumen formation, as branches were thickening, cells were gradually epithelializing and displayed an increasingly intense E-cadherin signal, particularly at the future apical side (Figures 4C and 4D). For branches more than two cells wide, we further found that the central cell population, potentially later eliminated through apoptosis, was surrounded by a strong E-cadherin signal (Figure 4E, Video S8 and Video S9).

Video S8. E-cadherin dynamics during epithelialisation E-cadherin signal in a PDAC organoid expressing endogeneous mNeonGreen E-cadherin during the Thickening phase (day 7–10), showing the progressive epithelialization of the system. Summed slice projection projection, imaged via confocal microscopy. Time-lapse imaging consists of an image taken every 30 min and is displayed at 20 fps. Time (hh:mm:ss). See also Figure 4.

Video S9. Central cell population shuttling in dark lumens Central population of to-be-eliminated cells being shuttled between epithelialized walls in a “dark lumen”, indicating that fluid and material can circulate within the organoid luminal network. Bright-field plane, drift-corrected. Time-lapse imaging consists of an image taken every 20 min, and is displayed at 20 fps. Time (hh:mm:ss). See also Figures 3 and 4.

Upon lumen nucleation, however, we found that rather than a complete disappearance of E-cadherin at the apical side, that could precede and enable the separation of walls due to a loss of adhesion, we observed an inhomogeneous distribution of the E-cadherin signal at the apical side, where cells either maintained an apical signal or individually lost it (Figure 4E). As lumens continued to grow in size, cells further lost their E-cadherin apical signal, but maintained expression both on the basal and lateral sides (Figure 4E). This observed progressive loss of E-cadherin at the apical side could be the result of recycling induced by the absence of cell-cell contact upon lumen opening,^23^ as well as the consequence of cadherin endocytosis mediated by members of the PKC protein family.^24^

To confirm the existence of an apico-basal identity that would support this asymmetrical distribution of E-cadherin, by defining molecularly an apical and a basal side,^9^ and allow directed processes such as protein transport, we investigated the distribution over time of PKC-ζ, an isoform of atypical protein kinase C (aPKC), known to be a key marker and player of the setting of polarity and the formation of lumens.^25^^,^^26^^,^^27^^,^^28^^,^^29^

Pre-lumen formation we could not detect any particular organization in the PKC-ζ signal (Figure 4E). However, as the first microlumens appeared, we detected a strong apical PKC-ζ signal (Figure 4E), which was maintained as lumens further grew (Figure 4E). This indicates that polarization is a process occurring in multiple steps, with actomyosin already being strongly localized at the apical side during the fault line formation stage, prior to cavity formation, whereas polarity defined by PKC-ζ appears concomitantly to lumen opening.

In this study, we sought to further assess the role of the actomyosin complex on lumen formation through perturbations with chemical compounds applied during the transition between the thickening and the lumen formation phases (Figures 4F–4J).

Using calyculin A (Thermofisher PHZ1044), a myosin phosphatase inhibitor, to increase cortical contractility,^21^^,^^30^^,^^31^ we observed, particularly on organoids with branches still elongating, that the extension stopped following drug addition, and that thin branches retracted and started forming bud-like structures (Figure 4H). As evidence of this increased contractility, we observed that the retraction of branches could lead to a rupture between cells at the tip, that remain attached to the collagen fibers at the front, and cells in the stem of the branch, that retract through the previously carved path in the collagen (Figure 4H). We, however, found that organoids largely remained capable of lumen formation upon exposure to calyculin A (Figures 4F, 4G, and S4A–S4A′).

Conversely, we used nitroblebbistatin (Santa Cruz Biotechnology sc-212797, hereafter “blebbistatin” in text and figures), a myosin II ATPase inhibitor, and ML7 (Santa Cruz Biotechnology sc-200557), a myosin light-chain kinase inhibitor, with the aim of interfering with myosin activity and decreasing cell contractility in organoids.^15^^,^^21^^,^^32^^,^^33^ While blebbistatin directly inhibits binding of non-muscle myosin II to actin, ML7 indirectly interferes with non-muscle myosin II by the inhibition of the myosin light-chain kinase activity, blocking the phosphorylation of the regulatory myosin light chains.^34^^,^^35^^,^^36^

Upon blebbistatin treatment we found a marked decrease in the lumen formation capability of organoids (Figures 4F and S4B), especially in the case of organoids which did not already display some cavities at drug addition time (Figures 4G and S4B’), highlighting the importance of myosin activity for lumen formation in PDAC organoids. Indicative of the changes in myosin contractility, we found that thinner branches could undergo fragmentation upon blebbistatin treatment, as cells could not maintain their elongated shape and switched to a rounded phenotype.

In contrast, we could not evidence an inhibitory effect on luminogenesis upon treatment with ML7, in the 10 μM–50 μM range, with the observed proportion of treated organoids not forming lumens being comparable to the one in untreated conditions (Figures 4F, 4G, and S4C–S4C′). These results demonstrate the possibility that other upstream factors, independent of the myosin light-chain kinase, are active during lumen formation in PDAC organoids, as it has been shown by previous studies investigating the RhoA activity in the development of the healthy pancreas.^33^ We, however, observed that treating organoids forming clear lumens with ML7 led to a rapid darkening of the cavities due to massive cell release and death at the apical side (Figure 4J), reminiscent of results reported in the context of endothelial luminogenesis perturbation.^37^

### PDAC organoids exhibit a progressive mucin expression upon lumen formation

**Figure 5:**
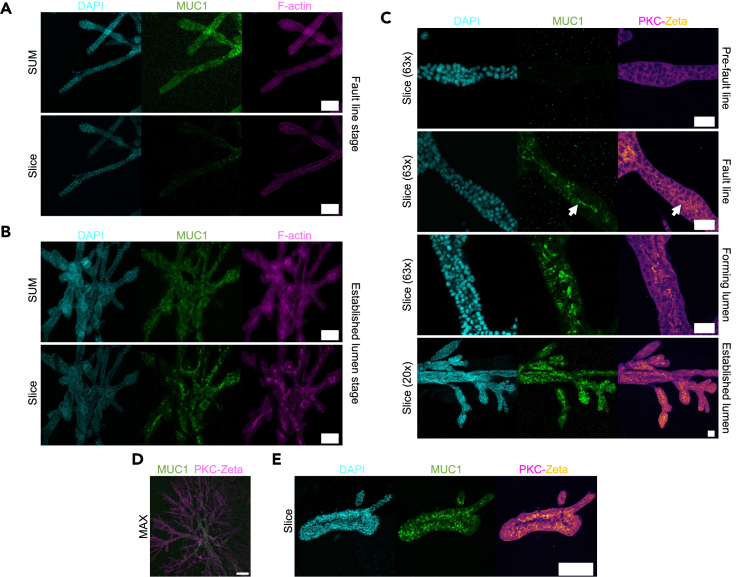
Mucin distribution (A) Staining of DAPI, Mucin (MUC1) and F-actin in an organoid pre-lumen formation, along a forming fault line. Top: summed slice z-projection. Bottom: z-slice. (B) Staining of DAPI, MUC1, and F-actin in an organoid with an established lumen. Top: summed slice z-projection. Bottom: z-slice. (C) Stainings of DAPI, MUC1, and PKC-ζ in organoids, from top to bottom: pre-fault line apparition, after fault line formation but pre-lumen formation, during lumen formation, and after lumen-formation, indicating the progressive apical polarization. (D) Staining of MUC1 and PKC-ζ indicating the restriction of strong MUC1 expression to the core region of an organoid where the lumen has formed. (E) Staining of DAPI, MUC1, and PKC-ζ in a cyst-like organoid, showing strong apical polarization and mucin expression. Scale bars: A and B 100 μm, C 50 μm, D and E 200 μm. Fluorescent images are all confocal-acquired and either slices, summed projections (SUM) or maximum intensity projections (MAX).

To further illustrate the interest of PDAC organoids as a system for luminogenesis study, we investigated the spatiotemporal distribution of MUC1, a mucin marking the lumens of pancreas’ precancerous lesions,^3^^,^^6^^,^^19^^,^^38^ the adult pancreas^19^ and the lumens of the developing embryonic pancreas,^25^^,^^39^ where it protects the epithelial lining and the luminal integrity.^40^ Immunofluorescence staining revealed that, prior to lumen formation, MUC1 appeared in the form of granule-like structures, seemingly following the epithelialization of cells, and the apparition of a strong F-actin central line (Figure 5A). Upon microlumen opening, we observed MUC1 expression at the apical side of microlumens, co-localized with the apical F-actin signal (Figure 5B), and correlated in space and time with the apparition of PKC-ζ, a pattern preserved up to the single lumen stage (Figure 5C). We noted that the expression of mucin on central side of the branches was restricted to areas where lumen had opened and polarized (Figure 5D), suggesting that cavity opening is a pre-requisite for central mucin accumulation. Organoids with a cyst-like phenotype likewise displayed polarized cavities with strong mucin expression on the apical side, reminiscent of large IPMN and MCN precancerous lesions^3^^,^^6^ (Figure 5E).

## Discussion

We have investigated the processes and the dynamics by which single seamless lumens emerge in PDAC organoids, and reported that both apoptosis-free and apoptosis-based luminogenesis mechanisms could be involved, either exclusively or together. While fluid intake-based mechanisms^13^^,^^21^^,^^22^ and cell death-based mechanisms^10^^,^^11^^,^^12^ have already been reported in the luminogenesis of other systems, including in the embryonic pancreas,^25^^,^^39^^,^^40^^,^^41^^,^^42^ the coexistence of the two mechanisms has rarely been observed within the same system and under the same culture conditions.

To study morphogenetic events—such as luminogenesis—and their potential significance in a pathological context, spheroid cultures have proven to be a powerful tool, capable of capturing important biological features of the organs they model, while presenting a number of experimental advantages such as reproducibility, ease-of-culture, or ease-of-imaging.^2^^,^^43^^,^^44^^,^^45^ However, their simplified geometry lacks resemblance to the actual *in vivo* structures, and prevents the observation of certain morphogenesis dynamics, such as the interaction of multiple lumens in a network-like environment established through branching. At the other end of the complexity spectrum, live *in vivo* imaging offers the closest insights to the actual physiological conditions, but comes at the cost of being technically difficult to perform and expensive to maintain, along with societal and regulatory pushes to refine, reduce or replace animal studies.^46^^,^^47^ Being architecturally more complex than spheroids but simpler than whole-animal studies, branched organoids thus represent a middle ground that possess enough architectural faithfulness to exhibit complex developmental dynamics while still remaining experimentally tractable.

The diversity in organoid structures, arising from the combination of stochasticity in self-organization and biophysical heterogeneity, allows the investigation of connections between variation in the dominating morphogenetic mechanisms and the emerging phenotypes.

At the molecular level, we found that the cellular rearrangements leading to an epithelial structure during the onset of lumen formation, appeared correlated with actomyosin dynamics in the organoids, pointing toward an important role for force generation.

Supporting this idea, perturbing myosin activity with blebbistatin (Figures 4F, 4G, 4I, and S4B–S4B′) led to a reduction in the lumen formation efficiency. Studies of luminogenesis in the developing embryonic murine pancreas,^33^ or in the zebrafish hepatopancreatic ductal system formation^15^ have evidenced that blebbistatin addition could indeed perturb proper lumen formation, causing disruptions in lumen connections, and gaps and loops in the luminal network. The specific action of myosin II in PDAC organoid luminogenesis, among its many identified roles,^48^^,^^49^^,^^50^^,^^51^ remains at this stage unclear. We note, however, that its reported function in driving cell shape changes and rearranging cells,^15^ may prove to be crucial in light of the epithelialization and polarization that we observed during the transition between the Thickening and the Lumen formation phases (Figures 4A–4E).

In untreated conditions, the respectively increasing apical F-actin and decreasing non-muscle myosin IIa signals (Figures 4A and 4B) point toward reduced actomyosin-generated stresses upon lumen opening and a stabilizing role for actin, maintaining tissue integrity upon the accumulation of fluid.^49^ Crucially though, whether in the case of calyculin, blebbistatin, or ML7 treatment, we did not observe the collapse of pre-existing lumens upon drug addition, suggesting that once lumens are established, cortical tension is not the sole component responsible for lumen shape stability, recapitulating results obtained in MDCK spheroids.^45^ The formation of epithelial walls surrounding the cavity, the pressure of the incorporated fluid, or other features such as a possible preferred apical domain size,^45^ could contribute to withstand those treatments, and thus represent promising targets for future perturbation studies.

Following lumen formation, we have evidenced the presence of basally or apically directed cell elimination events. We hypothesize that the elimination events in the epithelium post-lumen formation might be a way to maintain tissue homeostasis and result from the progressive epithelialization of the cells lining the lumen. Indeed, cells in the epithelium adopting a columnar-like morphology, and displaying an increased actin and E-cadherin signal—likely becoming stiffer in the process—while being surrounded by dense collagen, should lead to an environment which would mechanically prevent newly formed cells from remaining in the epithelium (Figures 4A, 4B, and 4E). Supporting this hypothesis, studies have reported that crowding could induce live cell extrusion, as a way to preserve a constant cell number.^52^^,^^53^ Moreover, dividing cells have been shown to generate protrusive forces upon mitosis^54^: however, if the forces are insufficient to deform the surrounding environment, mitosis will fail, and cells can either disassemble the mitotic spindle and reintegrate the chromosomes, or undergo apoptosis.^54^ Furthermore, at the molecular level, E-cadherin has been shown to be capable of forcing homeostatic conditions by reprogramming cell migration and cell cycle.^55^ This is in agreement with previously reported observations that Ki-67 expression, a marker for cell proliferation capability, decreased as organoids matured, disappearing first from the thick regions in the organoids cores, and persisting only in a few cells located in the buds.^8^ While the parameters governing the side of elimination of epithelium cells (basal or apical) remain unclear at this stage, models considering the interplay between cell-cell- and cell-ECM-adhesion,^56^ along with mechanosensitive responses^57^ have proposed mechanical control hypotheses that could be tested in further studies.

In addition, investigating whether the release of dark cells in cavities observed upon ML7 treatment (Figure 4J) stems from mechanical changes in the tissue induced by the myosin light-chain kinase inhibition and/or by the induction of apoptosis inside the luminal cavities^58^ could further shed some light on the process of apical cell elimination post-lumen formation that occurs in untreated conditions.

Lastly, comparing the development of the healthy embryonic pancreata with the one of organoids derived from cancer cells, has revealed a number of similarities but also of differences between the luminogenesis processes. We found that organoids appeared to form stochastically distributed microlumens, expressing proteins such as MUC1 at their apical side, exhibiting markers of apical polarity such as members of the PKC family, and undergoing fusion with each other to form a connected network, in a fashion similar to embryonic pancreata.^25^^,^^29^^,^^40^ In both cases, luminogenesis also appeared to be impaired when perturbing myosin II activity.^33^ Despite these similarities, however, PDAC organoids growth appeared to follow a timeline inverted compared to the one of the murine embryonic pancreas. Organoids first develop a branched structure and buds, before forming microlumens that coalesce into a seamless network.^8^ In contrast, the embryonic pancreas starts from the dorsal bud mass at E9.5, with a primary central lumen at E10-E10.5 and adjacent microlumens appearing at E10.5-E11.5 fusing to form the luminal plexus, only then followed by the apparition of tips and branches from E12.5 onwards.^29^^,^^40^ A further systematic comparison between the two systems could shed further insight on if and how cancer cells leverage biological mechanisms and pathways similar to those used during embryogenesis, when forming cancerous lesions.

For instance, to reveal whether polarization plays a role as an apical determinant and as a precursor to microlumen nucleation, as has been reported in the embryonic pancreas and elsewhere,^9^^,^^25^ future studies on PDAC organoids could focus on the recruitment dynamics of PKC-ζ and of other polarity-associated proteins such as Rab11.^59^

Furthermore, negatively charged mucins have been reported to be capable of initiating the separation of adjacent cell membranes through electrostatic repulsion, thereby contributing to the formation of a cavity, both in blood vessel vascular lumen formation^60^ and in mouse and human embryo lumen formation,^61^ which could constitute yet another lumen formation mechanism in PDAC organoids.

Our study thus demonstrates that branched organoids offer a powerful platform to study in space and time the simultaneous contributions of multiple luminogenesis mechanisms in a complex tubular network, a crucial step toward our understanding of the early events occurring in the precancerous lesions of PDAC, and the potential role played by lumens there.

### Limitations of the study

As in numerous self-organizing systems, the phenotypic heterogeneity spontaneously arising between organoids complicates the study of inter-sample similarities and differences, hampers normalization efforts, and hinders quantification, particularly as PDAC organoids possess a complex architecture. We are, however, hopeful that advances in the control of heterogeneity such as through the pre-patterning of the microenvironment, or through the enhanced selection of organoids according to specific transcriptomic profiles, may allow future works to overcome these difficulties. Furthermore, we had to strike a balance between fast high-content bright-field live-imaging—suffering from lower spatial resolution, particularly in the *z-*direction—and precise confocal live-imaging, which while well-resolved spatially, requires a slower scanning speed. While we are confident that our system can be scaled up, especially when engineering appropriate fluorescent markers for a desired readout, the availability of our microscopy platforms has restricted the number of samples we could analyze in this study, and the level of detail that we could reach for each of them. Lastly, we stress that while similarities were reported between *in vitro* organoids and *in vivo* cancerous or pre-cancerous lesions, further characterization is needed to fully establish to which extent cancer properties are recapitulated.

## STAR★Methods

### Key resources table

**Table:** 

REAGENT or RESOURCE	SOURCE	IDENTIFIER
**Antibodies**		

E-cadherin (24E10) Rabbit mAb	Cell Signaling Technology	Cat#: 3195, RRID: AB_2291471
PKC-Zeta antibody (H-1)	Santa Cruz Biotechnology	Cat# sc-17781 AF647, RRID: AB_628148
Phospho-Myosin Light Chain 2 (Thr18/Ser19) (E2J8F) Rabbit mAb	Cell Signaling Technology	Cat#: 95777
Anti-MUC1 antibody	Abcam	Cat#: ab15481, RRID: AB_301891
Goat anti-Rabbit IgG (H+L) Highly Cross-Adsorbed Secondary Antibody, Alexa Fluor™ 488	Invitrogen	Cat#: A11034, RRID: AB_2576217

**Bacterial and virus strains**		

Competent cells XL-1 blue	Agilent	Cat#: 200249

**Chemicals, peptides, and recombinant proteins**		

Dulbecco’s Modified Eagle’s Medium – high glucose	Sigma-Aldrich	Cat#: D6429
Fetal Bovine Serum	Sigma-Aldrich	Cat#: F7524
Penicillin-Streptomycin	Sigma-Aldrich	Cat#: P4333
0.05 % Trypsin-EDTA	Sigma-Aldrich	Cat#: T3924
Polybrene Infection / Transfection Reagent	Sigma-Aldrich	Cat#: TR-1003-G
Geneticin Selective Antibiotic (G418 Sulfate)	Sigma-Aldrich	Cat#: 10131035
Corning Collagen Type I, rat tail	Corning	Cat#: 354236
Calyculin A	Invitrogen	Cat#: PHZ1004
S-(-)-7-Desmethyl-8-nitro Blebbistatin	Santa Cruz Biotechnology	Cat#: sc-212797
ML-7 hydrochloride	Santa Cruz Biotechnology	Cat#: sc-200557
Ouabain	Biotechne/Tocris	Cat#: 1076
Alexa Fluor™ 488 Phalloidin	Invitrogen	Cat#: A12379
Alexa Fluor™ 633 Phalloidin	Invitrogen	Cat#: A22284
Paraformaldehyde	Sigma-Aldrich	Cat#: 158127
Triton™ X-100	Sigma-Aldrich	Cat#: T8787
Roti PreMix PBS	Carl Roth	Cat#: 0890.1
Goat serum donor herd	Sigma-Aldrich	Cat#: G6767
Donkey serum	Sigma-Aldrich	Cat#: D9663
Bovine Serum Albumin (BSA), Fraction V	Carl Roth	Cat#: 8076.2
NucView 488 Caspase-3 Substrate	Biotium Science™	Cat#: 10402
NucView 530 Caspase-3 Substrate	Biotium Science™	Cat#: 10406
Dextran, Alexa Fluor™ 488; 3000 MW, Anionic	Invitrogen	Cat#: D34682
SiR-actin kit	Spirochrome	Cat#: SC001
SOC Outgrowth Medium	New England Biolabs	Cat#: B9020L
NEBuilder HiFi DNA Assembly Master Mix	New England Biolabs	Cat#: E2621
Agarose Standard	Carl Roth	Cat#: 3810.3
BamHI-HF	New England Biolabs	Cat#: R3136
SalI-HF	New England Biolabs	Cat#: R3138

**Critical commercial assays**		

Mirus TransIT-X2 Dynamic Delivery System	VWR (Mirus Bio)	Cat#: MIRUMIR-6000
Platinum™ SuperFi Green PCR Mastermix	Invitrogen	Cat#: 12369050
Monarch DNA Gel Extraction Kit	New England Biolabs	Cat#: T1020L
Qiagen Plasmid Kit	Qiagen	Cat#: 12162

**Deposited data**		

Source data	This paper	https://doi.org/10.5281/zenodo.11060358
Analysis code	This paper	https://doi.org/10.5281/zenodo.11060358

**Experimental models: Cell lines**		

Phoenix ECO	Swift, S. et al. 2001.	https://doi.org/10.1002/0471142735.im1017cs31
PDAC 9591	Mueller S. et al., 2018.	https://doi.org/10.1038/nature25459
Cdh-1 mNG PDAC 9591	Dieter Saur	N/A
MyosinII-GFP PDAC 9591	This paper	N/A

**Oligonucleotides**		

CMV-FWD: 5’-GGAGTTCCGCGTTACATAACTTACG-3’	Integrated DNA Technologies IDT	
CMV-REV: 5’-GTTCACTAAACCAGCTCTGCTTATAT-3’	Integrated DNA Technologies IDT	
MyosinIIA-FWD: 5’-CTACCGGTCGCCACCATGG-3’	Integrated DNA Technologies IDT	
MyosinIIA-REV: 5’-CTGATTATGATC AGTTATCTAGAAGCG-3’	Integrated DNA Technologies IDT	
IRES-FWD: 5’-CCGTCTTTTGGCAATGTGAGGG-3’	Integrated DNA Technologies IDT	
IRES-REV: 5’-TTATCATCGTGTTTTTCAAAGGAAA-3’	Integrated DNA Technologies IDT	
Neomycin-FWD: 5’-ATGATTGAACAAG ATGGATTGCACGC-3’	Integrated DNA Technologies IDT	
Neomycin-REV: 5’-CCCCAGAGTCCCGCTCAGAAG-3’	Integrated DNA Technologies IDT	

**Recombinant DNA**		

pTK93_Lifeact-mCherry	Iain Cheeseman	Plasmid #46357, http://n2t.net/addgene:46357, RRID: Addgene_46357
mRuby-LC-Myosin-N-7	Michael Davidson	Plasmid #55871, http://n2t.net/addgene:55871, RRID: Addgene_55871
MyosinIIA-GFP	Matthew Krummel	Plasmid #38297, http://n2t.net/addgene:38297, RRID: Addgene_38297
pQCXIP-mCherry-Halo-YAP1	Yutaka Hata	Plasmid #128336, http://n2t.net/addgene:128336, RRID: Addgene_128336

**Software and algorithms**		

Geneious Prime (ver. 2023.0.4)	Geneious	https://www.geneious.com/ RRID: SCR_010519
LAS X (ver. 3.5.7.23225)	Leica Microsystems	RRID: SCR_013673
LAS X (ver. 3.7.5.24914)	Leica Microsystems	RRID: SCR_013673
ImageJ (ver. 1.54f)	Johannes Schindelin	https://imagej.net/ij/ RRID: SCR_003070
TurboReg plugin	Philippe Thévenaz	http://bigwww.epfl.ch/thevenaz/turboreg/ RRID: SCR_014308
StackReg plugin	Philippe Thévenaz	https://bigwww.epfl.ch/thevenaz/stackreg/
HyperStackReg plugin	Ved Sharma	https://doi.org/10.5281/zenodo.2252521
KymoResliceWide plugin	Eugene Katrukha	https://doi.org/10.5281/zenodo.4281086
Bio-Formats	Melissa Linkert	https://doi.org/10.1083/jcb.201004104
Arivis Vision4D (ver. 4.0.0)	Zeiss	https://www.arivis.com/arivis-news/arivisvision4d40 RRID: SCR_018000
Python (ver. 3.7.10)	Python Software Foundation	https://www.python.org/ RRID: SCR_008394
Inkscape v1.3	Inkscape	https://inkscape.org/en/ RRID: SCR_014479

### Resource availability

#### Lead contact

Further information and requests for resources should be directed to, and will be fulfilled by, the lead contact, Andreas R. Bausch (abausch@mytum.de).

#### Materials availability

This study did not generate new unique reagents.

#### Data and code availability

• Source data used to generate the graphs in this manuscript have been deposited at a Zenodo repository and are publicly available as of the date of publication. DOIs are listed in the key resources table. Microscopy data reported in this paper will be shared by the lead contact upon request.
• All original code has been deposited at a Zenodo repository and is publicly available as of the date of publication. DOIs are listed in the key resources table.
• Any additional information required to reanalyse the data reported in this paper is available from the lead contact upon request.

### Experimental model and study participant details

#### Cell cultures

Primary tumour cells (PDAC) were a gift by the laboratories of Prof. Maximilian Reichert and Prof. Dieter Saur (Technical University of Munich). Cells were authenticated by genotyping. The Phoenix ECO helper-free retrovirus producer cell line was kindly gifted by the laboratory of Prof. Carsten Grashoff (Universität Münster). All cells were tested and confirmed negative for mycoplasma contamination every 6 months.

### Method details

#### Cell and organoid culture

As described in ref.^8^^,^^62^, primary tumour cells derived from a ${Ptf1a}^{Cre/+}$*;* ${Kras}^{G12D/+}$ mouse were first seeded in 75 cm^2^ flasks with Dulbecco’s Modified Eagle’s Medium (DMEM) - high glucose (Sigma D6429) supplemented with 10% v/v Fetal Bovine Serum (FBS) (Sigma F7524), with medium being fully exchanged every 2 to 3 days, and cultured in an incubator with an 80% humidity and 5% CO_2_ atmosphere, at 37°C. At confluence, cells were passaged using 0.05% Trypsin-EDTA (Sigma T3924).

Identically to PDAC cells, Phoenix ECO helper-free retrovirus producer cell line (hereafter ”Phoenix ECO cells” in text and figures)^63^ were cultured with DMEM - high glucose (Sigma D6429) supplemented with 10% v/v FBS and 1% Penicillin-Streptomycin (Sigma P4333) in an incubator with an 80% humidity and 5% CO_2_ atmosphere, at 37°C.

For the stable expression of fluorescent markers, retroviral transduction as well as CRISPR/Cas9 transfections were performed with the same primary tumour cells. Retroviral transfection of PDAC cells was implemented during a 8 day protocol using a generated retroviral plasmid for GFP-tagged non-muscle myosin IIa. At day 1, Phoenix ECO cells were seeded in a 175 cm^2^ flasks. Having reached a confluency of 50 to 60% (day 2), Phoenix ECO cells were transfected using Mirus TransIT-X2 Dynamic Delivery System (VWR MIRUMIR-6000) as described in the manufacturer protocol. Media exchange was performed after 24 hours (day 3) of incubation. Simultaneously, PDAC cells were seeded in a 75 cm^2^ flask. Virus was harvested after 48 hours (day 4) from Phoenix ECO cells and sterile filtered (0.45 μm pore size). Supplemented with 7.5 μg/ml polybrene (Sigma TR-1003-G) the virus conditioned media was added to the PDAC cells and incubated for 24 hours. This was repeated at day 5. After 48 hours of incubation with virus conditioned media, media was exchanged for PDAC cells with fresh DMEM - high glucose (Sigma D6429) supplemented with 10% v/v FBS (Sigma F7524) and 1% Penicillin-Streptomyosin (Sigma P4333). After 72 hours cells were passaged as described above. Selection of fluorescently labeled cells was implemented by antibiotic resistance, using Geneticin Selective Antibiotics (Gibco 10131035) and FACS sort with BD Aria Fusion.

A CRISPR/Cas9 system was used to endogeneously label E-cadherin in the used primary tumour cells.

To prepare organoids, upon passaging, the cell suspension was diluted to reach a concentration of 500 cells/mL medium, and mixed gently with cell culture medium, neutralising solution (550 mM HEPES in 11x PBS) and Collagen Type I (rat tail, 354236 Corning) to reach a final collagen concentration of 1.3 mg/mL. The gel was incubated at 37°C for 1h30 to polymerise, before being detached from the culture plate, and covered in additional cell culture medium. Organoids were cultured in ibidi μ-Slide 2-wells (80286), ibidi μ-Plate 24 Well (82426) or Sarstedt 24-well (83.3922).

#### Imaging

Confocal imaging was performed on a Leica DMi8 confocal microscope (software LAS X version 3.5.7.23225), and bright field imaging was performed on a Leica Thunder DMi8 Thunder microscope (software LAS X version 3.7.5.24914). For live sample imaging, samples were kept at 37°C and under a 5% CO_2_ atmosphere using an ibidi Stage Top Incubation System (ibidi 10722).

For live-cell imaging of actin, organoids were stained with 1 nM of SiR-actin (Spirochrome SC001) overnight before starting the measurement during the period spanning the Thickening and the Lumen formation phases. To additionally detect apoptotic cells, organoids were co-labelled using a fluorescent caspase-3/7 activity reporter (Biotium #10402 and #10406) and incubated for at least 30 minutes prior to image acquisition (see Table S3).

To visualise fluid intake, dextran (Invitrogen D34682) was added to cell culture media at a concentration of 200 μg/ml before the measurement.

#### Drug treatment

For drug treatment experiments, organoids were cultured in μ-Plate 24-wells (ibidi 82426). Organoids were live imaged for approximately 24 hours prior to drug addition, to control for the presence of the hallmarks of the desired developmental phase to perturb, before introducing the drug to the culture well and resuming the live imaging. The presence of lumens in organoids after ouabain (Tocris 1076), calyculin (Invitrogen PHZ1044), blebbistatin (Santa Cruz SC-212797) and ML7 (Santa Cruz sc-200557) treatment was determined by visually assessing the planes of bright field z-stacks recordings over time. We recorded whether organoids were displaying cavities prior to drug addition, after drug addition, as well as the type of lumens predominantly formed (clear or dark). In Figures 2, 4, S1, and S4, we excluded from our analysis the organoids that were fragmented or did not exhibit hallmarks of the thickening to Lumen formation phase with thick and budding branches (e.g. organoids with very thin branches remaining in an extension phase-like phenotype). Organoids that reached a phenotype that could allow lumen formation following drug addition, were included in the analysis. Based on our criteria, organoids were observed for two to three days following drug addition to quantify the lumen formation capability. The “No treatment” data shown in Figures 4F and 4G pools together the untreated organoids shown in Figures S4A–S4C’.

#### Immunofluorescent staining

Organoids were washed and fixed using 4% paraformaldehyde (Sigma 158127) for 15 minutes at room temperature (RT). Cells were permeabilised using 0.2% Triton X-100 (Sigma T8787) in PBS (Roth 0890.1) for 10 minutes at RT and blocked overnight at 4°C using 10% goat (Sigma G6767) or donkey serum (Sigma D9663)/0.1% Bovine Serum Albumin (BSA) (Roth 8076.2). Cells were then labelled with primary antibodies (see Table S1) diluted in 0.1% BSA and incubated overnight at 4°C as follows: E-cadherin [24E10] (Cell Signaling 3195S, diluted 1:50), PKC-Zeta (Santa Cruz Biotechnology, diluted 1:50), phosphorlyated myosin light chain 2 [E2J8F] (Cell Signaling 95777S, diluted 1:200) and mucin 1 (Abcam ab15481, diluted 1:150). Afterwards, secondary antibody (Invitrogen A11034, diluted 1:250) and phalloidin (Invitrogen A12379 and A22284, diluted 1:250) diluted in 0.1% BSA were added and incubated at least two hours at RT in the dark (see Tables S2 and S3).

#### Cloning

The GFP-tagged non-muscle myosin IIa plasmid for transfection was generated using restriction enzymes and PCR reaction to amplify the fragments for Gibson assembly. The backbone was digested for 2 hours at 37°C using BamHI (New England Biolabs R3136) and SalI (New England Biolabs R3138) in combination with the plasmid pTK93_Lifeact-mCherry, which was a gift from Iain Cheeseman (Addgene plasmid # 46357).^64^ The PCR reactions were performed with the Platinum SuperFi Green PCR Mastermix (Invitrogen 12369050) as described in the manufacturer protocol with the following plasmids and primers. The CMV promoter was extracted from mRuby-LC-Myosin-N-7,a gift from Michael Davidson (Addgene plasmid # 55871), the myosinIIA-GFP from Myosin-IIA-GFP was a gift from Matthew Krummel (Addgene plasmid # 38297),^65^ the IRES sequence from pQCXIP-mCherry-Halo-YAP1 was a gift from Yutaka Hata (Addgene plasmid # 128336),^66^ and the Neomycin resistance from mRuby-LC-Myosin-N-7 was a gift from Michael Davidson (Addgene plasmid # 55871).

The PCR primers were used:

CMV promoter:

Forward 5’-GGAGTTCCGCGTTACATAACTTACG-3’

Reverse 5’-GTTCACTAAACCAGCTCTGCTTATAT-3’

Myosin-IIA-GFP:

Forward 5’-CTACCGGTCGCCACCATGG-3’

Reverse 5’-CTGATTATGATCAGTTATCTAGAAGCG-3’

IRES:

Forward 5’-CCGTCTTTTGGCAATGTGAGGG-3’

Reverse 5’-TTATCATCGTGTTTTTCAAAGGAAA-3’

Neomycin:

Forward 5’-ATGATTGAACAAGATGGATTGCACGC-3’

Reverse 5’-CCCCAGAGTCCCGCTCAGAAG-3’

DNA fragments were separated by gel electrophoresis using a 1% agarose gel (Roth 3810.3). Selected fragments were extracted from the gel using the Monarch DNA Gel Extraction Kit (New England Biolabs T1020L) according to the manufacturer protocol. The Gibson assembly was performed using the NEBuilder HiFi DNA Assembly Master Mix (New England Biolabs E2621) with a molar ratio of insert to backbone of 2 and incubated for 60 minutes at 50°C. For the transformation of competent cells with the Gibson assembly product, a XL-1 blue bacterial strain (Agilent 200249) was incubated for 30 minutes on ice with 2 μl of the product. Afterwards cells get heat shocked for 40 seconds at 42°C and again incubated on ice for 10 minutes, before being incubated for 1 hour at 37°C with SOC media (New England Biolabs B9020L). Cells were centrifuged at 3500 xG for 5 minutes, plated out on agar plates and incubated at 37°C overnight. Colonies were selected and 250 ml overnight culture was prepared to isolate the plasmid DNA according to the manufacturer protocol (Qiagen 12162). Sequencing results were analysed using Geneious Prime (ver. 2023.0.4).

### Quantification and statistical analysis

#### Identification of organoids’ developmental phase

Due to the phenotypical heterogeneity between the samples, we assessed the developmental phases heuristically by combining the number of days since seeding with a number of qualitative features described in a previous study.^8^ For the extension phase, we considered organoids mostly between days 6 and 10 post-seeding that displayed a pattern of branching and ballistic-like elongation, with branches displaying a spiky protrusion at their tip. For the thickening phase, we considered organoids mostly between days 9 and 12 post-seeding that displayed retracting branches, losing their spiky protrusions and rounding into terminal end bud-like structures, and displaying an increase in width. For the lumen formation phase, we considered organoids mostly between days 11 and above that displayed the formation of microcavities, whether clear or dark (see Figures 2 and 3).

#### Lumen type identification

To determine the type of lumens formed in organoids, bright field optical stacks of live-imaged organoids were analysed, with multiple focal planes reviewed. To discriminate between lumens nucleating via central cell apoptosis (dark lumens) and lumens nucleating via fluid intake (clear lumens) later filled with post-lumen formation cell elimination - which can both appear as dark cavities in bright field - live imaging was started during the thickening phase of the majority of the organoids, pre-lumen formation, to monitor the entire lumen formation process.

#### Image and data analysis

Images were analysed using ImageJ (ver. 1.54f),^67^ the Bio-Formats plugin for ImageJ (ver. 8.0.0)^68^ and Arivis Vision4D (ver. 4.0.0). Images were drift corrected using the TurboReg, StackReg^69^ and HyperStackReg^70^ plugins for ImageJ.

Python (ver. 3.7.10) and the *seaborn* package were used to analyse numerical data and produce graphs. Linear regression fits were performed using the Python *stats* linregress function. Box plots indicate the interquartile range (IQR, rectangle), the median (line), and 1.5 times the IQR (whiskers). Images were assembled using Inkscape v1.3.

#### Intensity profiles and kymographs

Line intensity profiles were obtained using the “Plot Profile” tool of ImageJ on z-slice images, following a thresholding step.

For kymographs, z-stacks were first z-projected as Sum Slices projections and drift-corrected. A 100 μm long and 50 μm wide line was then drawn in the region of interest and kymographs were obtained using the KymoResliceWide plugin for ImageJ^71^ using the “Intensity across width: Average” and “Ignore image calibration” options selected.

#### Lumen morphometrics

To quantify the evolution of clear lumen geometries over time, we looked for the middle cross-sectional plane of lumens on bright field z-stacks and manually segmented the lumens using the Polygon tool from ImageJ.

Due to inter- and intra-organoid heterogeneity, cavities could start forming at different timepoints between and within organoids. We labelled cavities that we could segment from their nucleation point onward as “Nuc” (for “nucleating”), whereas cavities that had already nucleated and grown before the start of imaging were labelled as “Swe” (for “swelling”). In case a tracked microlumen fused with a neighbouring cavity, we recorded the event and labelled it additionally as “fus” (for “fusing”). In the case of organoids displaying a “blowing up” phenotype, we delineated virtual boundaries around swelling cavities to capture the increase in the minor axis, even in the case of fusing or connected cavities. This results in an under-estimation of the real area of blowing up lumens.

Swelling rates were approximated through linear regression, excluding tracks that contained fusion events between microlumens.

#### Apoptotic event counting

Apoptotic events were detected using Arivis Vision4D *Blob Finder* routine on the fluorescent signal. Rates of apoptosis were estimated by fitting a linear function to the cumulated apoptosis events detected curves. The apoptosis event counting was limited to 24 hours to avoid as much as possible counting the cell death events occurring post-lumen formation.
