# Supplementary material for: Coexisting mechanisms of luminogenesis in pancreatic cancer-derived organoids
Source: iScience. 2024 Jun 18;27(7):110299. doi: 10.1016/j.isci.2024.110299 (PMC11269295; doi:10.1016/j.isci.2024.110299)
Supplement: Document S1. Figures S1–S4 and Tables S1–S3 [file mmc1.pdf]

iScience, Volume *27*

Supplemental information

Coexisting mechanisms of luminogenesis in pancreatic cancer-derived organoids

Samuel J. Randriamanantsoa, Marion K. Raich, Dieter Saur, Maximilian Reichert, and Andreas R. Bausch

Supplementary Figure S1: Supporting measurements for clear and dark lumens, Related to Figure 2, 3

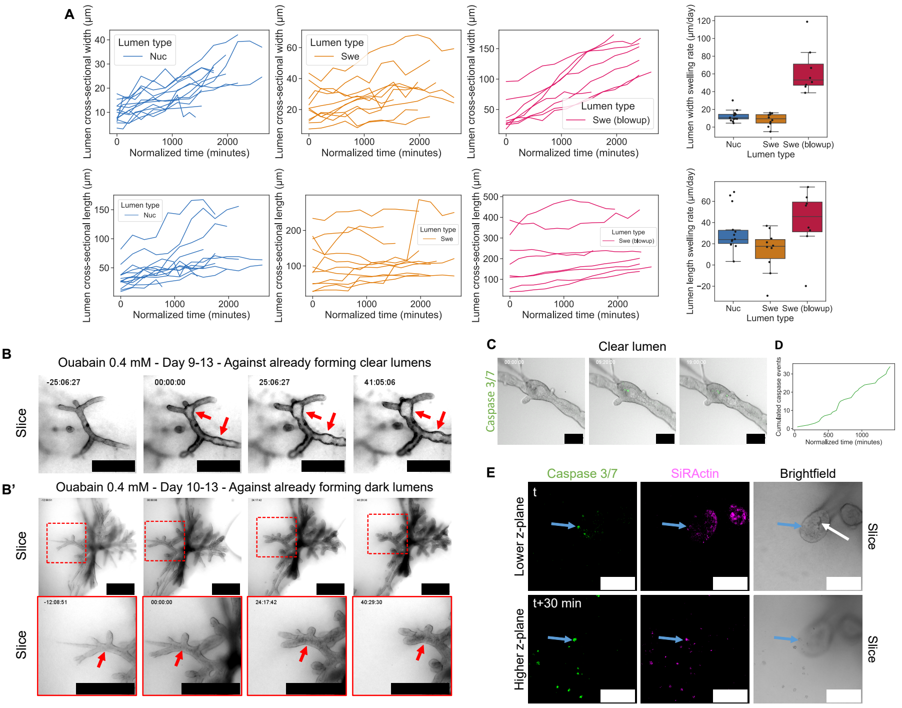

Fig. S1 Supporting measurements for clear and dark lumens. A For the clear lumens tracked in Fig. 2, evolution of the cavities width (minor axis of the fitted polygon, top), and length (major axis of the fitted polygon, bottom), and corresponding box plots of the estimated rates of change ("Nuc": n = 14 lumens, N = 5 organoids; "Swe": n = 11 lumens, N = 4 organoids; "Swe (blowup)": n = 8 lumens, N = 3 organoids). Box plots indicate the interquartile range (IQR, rectangle), the median (line), and 1.5 times the IQR (whiskers). Treatment during the Thickening to Lumen formation phase with 0.4 mM ouabain may fail to prevent lumen formation and development for two phenotypes: in **B**, organoids that had already nucleated clear lumens appear to be able to continue increasing their size; in **B'**, organoids that had already initiated the formation of dark lumens can continue their apoptosis process (bottom row shows a close up view of a dark lumen continuing its formation even after ouabain addition). Drug addition was considered as timepoint 00:00:00, and negative time values denote periods prior to drug addition. **C** Time-lapse of a clear lumen in an organoid labelled with NucView Caspase-3 Enzyme Substrate, showing very few apoptotic events occurring. Confocal slices. The corresponding cumulated counting of apoptotic events is shown in **D**. **E** Example of basal-side elimination shown in Fig. 3E, with apoptotic events, F-actin and bright field channels being shown. The blue arrow tracks a cell being expelled on the basal side, while the white arrow indicates an apical lumen. This implies that the presence of an apical lumen does not systematically force all elimination events to occur towards the apical side. Scale bars: **B, B'** 500 µm; **C, E** 100 µm. Fluorescent images in **D, G**: confocal slices. Bright field images in **F, G**: planes.

Supplementary Figure S2: Clearing of cells in cavities, Related to Figure 3

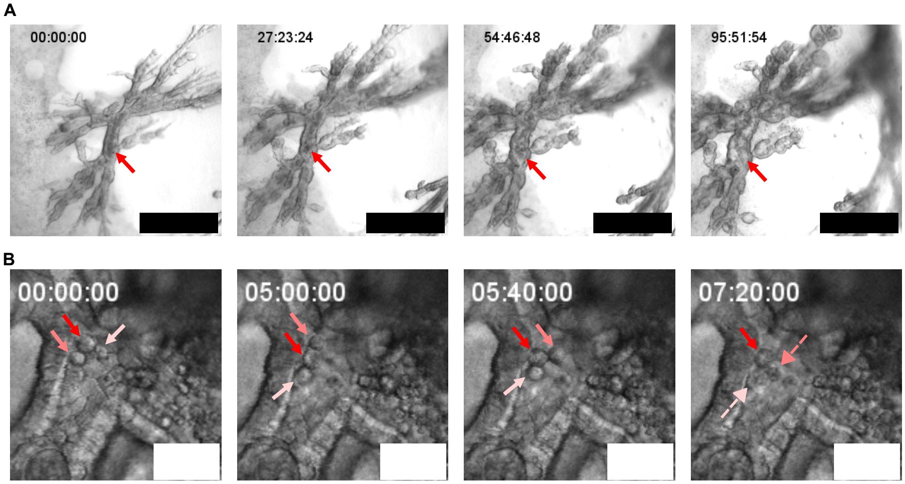

Fig. S2 Clearing of cells in cavities. A Dark lumens filled with apoptotic cells become progressively clearer over time as fluid is further incorporated in the cavities, and as floating cells degrade. **B** Closeup view of a cavity with floating cells inside, marked by solid arrows. As fluid circulates through the cavity, cells are being displaced. In the last panel, two of the tracked cells, marked by dashed arrows undergo degradation, with their membranes losing their integrity. Scale bars: **A** 500 µm, **B** 50 µm.

Supplementary Figure S3: Actin and myosin stainings, Related to Figure 2, 3, 4

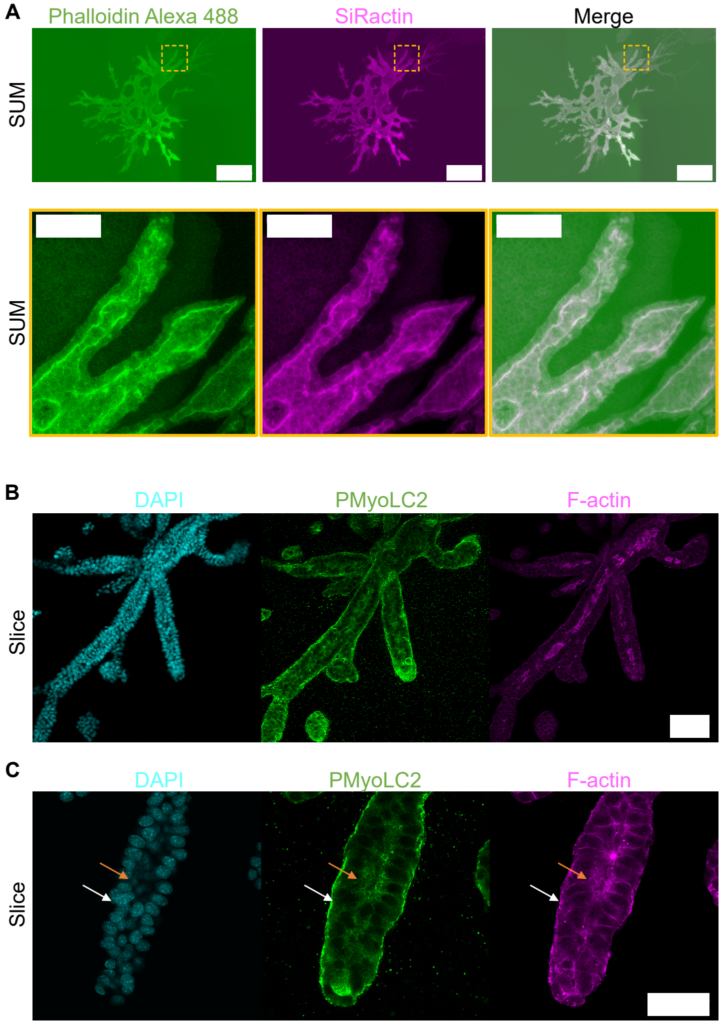

Fig. S3 Actin and myosin stainings. A Top: Organoid co-stained with Phalloidin Alexa 488 (green) and SiR-actin (magenta) exhibiting excellent overlap between the two signals. Bottom: closeup view of terminal structures. Both top and bottom panels are confocal summed slice projections. **B** Staining of nuclei (DAPI), Phosphorylated Myosin Light Chain 2 (PMLC2) and F-actin in an organoid displaying nucleating lumens. **C** Closeup view of terminal structures . The white arrow indicates the basal side and the orange arrow indicates the apical side. The PMLC2 apical signal spatially correlate with the non-muscle myosin IIa signal. **B** and **C** are confocal slices. Scale bars: **A**(top) 500 µm, **A**(bottom) and **B** 100 µm, **C** 50 µm.

Supplementary Figure S4: Perturbation of actomyosin dynamics, Related to Figure 4

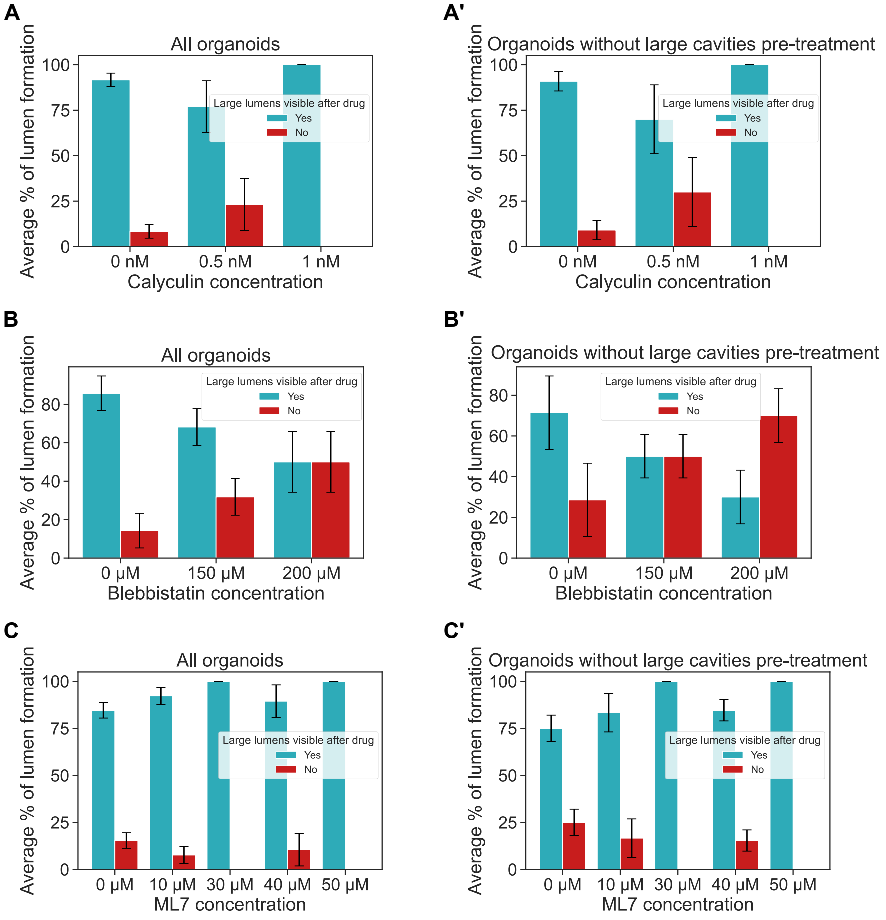

Fig. S4 Perturbation of actomyosin dynamics Average percentage of lumen formation in organoids treated at the Thickening-to-Lumen formation phase with blebbistatin, ML7 or calyculin (resp. **A**, **B**, **C**) at various concentrations. Both organoids with and without large cavities prior to drug addition are quantified in **A**, **B** and **C**. A similar analysis restricted to organoids that did not display large lumens before drug addition is shown in **A'**, **B'** and **C'**. Sample sizes: **A** Calyculin - 0 nM : N = 12 ; 0.5 nM : N = 13 ; 1 nM : N = 8 organoids. **A'** Calyculin - 0 nM : N = 11 ; 0.5 nM : N = 10 ; 1 nM : N = 7 organoids. **B** Blebbistatin - 0 µM : N = 14 ; 150 µM : N = 22 ; 200 µM : N = 14 organoids. **B'** Blebbistatin - 0 µM : N = 7 ; 150 µM : N = 14 ; 200 µM : N = 10 organoids. **C** ML7 - 0 µM : N = 13 ; 10 µM : N = 12 ; 30 µM : N = 20 ; 40 µM : N = 19 ; 50 µM : N = 12 organoids. **C'** ML7 - 0 µM : N = 8 ; 10 µM : N = 6 ; 30 µM : N = 5 ; 40 µM : N = 13 ; 50 µM : N = 3 organoids. Error bars indicate the standard error of the weighted mean.

Supplementary Materials - Tables

Epitope [Clone]	Conjugation	Host	Catalogue #	Supplier	Dilution
E-cadherin [24E10]	-	Rabbit	3195S	Cell Signaling	1:50
PKC-Zeta	Alexa 647	Mouse	sc17781	Santa Cruz Biotechnology	1:50
Phosphorylated Myosin Light Chain 2 [E2J8F]	-	Rabbit	95777S	Cell Signaling	1:200
Mucin 1	-	Rabbit	ab15481	Abcam	1:150

Table S1 Primary antibodies, Related to Figure 4, 5, S3.

Host/Isotype	Species reactivity	Conjugation	Catalogue #	Supplier	Dilution
Goat	Rabbit	Alexa 488	A11034	Invitrogen	1:250

Table S2 Secondary antibodies, Related to Figure 4, 5, S3.

Name	Target	Conjugation	Catalogue	Supplier	Dilution
Phalloidin	F-actin	Alexa 488	A12379	Invitrogen	1:250
Phalloidin	F-actin	Alexa 633	A22284	Invitrogen	1:250
SiR-actin	F-actin	Silicon Rhodamine	SC001	Spirochrome	100 nM
NucView 488 Caspase-3 Substrate	Caspase-3/7 activity	NucView 488 dye	10402	Biotium	5 µM
NucView 530 Caspase-3 Substrate	Caspase-3/7 activity	NucView 530 dye	10406	Biotium	5 µM

Table S3 Additional fluorescent probes, Related to Figure 2, 3, 4, 5, S1, S3 .
